# Medicinal Prospects of Antioxidants From Algal Sources in Cancer Therapy

**DOI:** 10.3389/fphar.2021.593116

**Published:** 2021-03-05

**Authors:** Umme Tamanna Ferdous, Zetty Norhana Balia Yusof

**Affiliations:** ^1^ Aquatic Animal Health and Therapeutics Laboratory (AquaHealth), Institute of Bioscience, Universiti Putra Malaysia, Selangor, Malaysia; ^2^ Faculty of Biotechnology and Biomolecular Sciences, Department of Biochemistry, Universiti Putra Malaysia, Selangor, Malaysia; ^3^ Bioprocessing and Biomanufacturing Research Center, Universiti Putra Malaysia, Selangor, Malaysia

**Keywords:** algae, antioxidant, cancer therapy, reactive species, dietary supplements, cancer

## Abstract

Though cancer therapeutics can successfully eradicate cancerous cells, the effectiveness of these medications is mostly restricted to several deleterious side effects. Therefore, to alleviate these side effects, antioxidant supplementation is often warranted, reducing reactive species levels and mitigating persistent oxidative damage. Thus, it can impede the growth of cancer cells while protecting the normal cells simultaneously. Moreover, antioxidant supplementation alone or in combination with chemotherapeutics hinders further tumor development, prevents chemoresistance by improving the response to chemotherapy drugs, and enhances cancer patients’ quality of life by alleviating side effects. Preclinical and clinical studies have been revealed the efficacy of using phytochemical and dietary antioxidants from different sources in treating chemo and radiation therapy-induced toxicities and enhancing treatment effectiveness. In this context, algae, both micro and macro, can be considered as alternative natural sources of antioxidants. Algae possess antioxidants from diverse groups, which can be exploited in the pharmaceutical industry. Despite having nutritional benefits, investigation and utilization of algal antioxidants are still in their infancy. This review article summarizes the prospective anticancer effect of twenty-three antioxidants from microalgae and their potential mechanism of action in cancer cells, as well as usage in cancer therapy. In addition, antioxidants from seaweeds, especially from edible species, are outlined, as well.

## Introduction

Oxygen is essential to aerobic life conditions and represents the main driving force for the maintenance of cell metabolism and viability. Simultaneously, oxygen also has a potential hazard due to its paramagnetic characteristics stimulating the formation of partially oxidized high reactive components, known as reactive oxygen species (ROS) (Francenia Santos-Sánchez et al., 2019). Though the metabolism of oxygen produces ROS in living organisms as by-products, they have a significant influence on cell signaling and redox homeostasis. Sometimes, ROS levels can be increased upon contacting with exogenous or endogenous sources, rendering a stress condition in the cell that is called oxidative stress. In such a state, the ROS level reaches a toxic threshold, and it manages to overcome the antioxidant system of the cell, thus escapes to elimination and remain in the cell. (Raza et al., 2017). These ROS give rise to negative oxidative stress that engenders some drastic changes in cellular function and metabolism through altering cellular signaling pathways, initiating genomic instability, or activating immunosuppression, which leads to carcinogenesis (Morry et al., 2017). Cancer cells are more sensitive to therapeutic drugs that produce excessive amounts of ROS or impair ROS scavenging capacity of cells, which provokes apoptosis (Mut-Salud et al., 2015).

Among a variety of treatments, chemotherapy remains the first choice of cancer treatment. Though drugs used in chemotherapy can successfully eliminate fast-growing cancerous tissues, these drugs can affect the mucous membranes of various organs. As a consequence, several side effects are noticed in cancer patients, such as anaphylaxis, a different type of cytopenia, toxicity to liver, heart, nephron, ear, and also nausea, vomiting, pain, diarrhea, alopecia, anorexia, cachexia, inflammation in mucous membranes, and asthenia (Oun et al.,2018). To compensate for these adverse effects, antioxidant supplements are often prescribed, which can help to ameliorate side effects while not affecting treatment efficacy (Ambrosone et al., 2019). Cancer survivors often consume vitamins or minerals supplements, natural plant-based products, or herbal medicines to alleviate the therapy-related side effects. The most common recommended antioxidants are vitamins, polyphenols, and carotenoids. Edible vegetables and fruits are an excellent reservoir of different antioxidant phytochemicals with varied antioxidant capacity and it has been recommended that intake of >400 g fruits and vegetables can prevent certain types of cancer (Miller and Snyder, 2012; Chester et al., 2019; Wall-Medrano and Olivas-Aguirre, 2020).

Besides these plant products, microalgae can be an excellent alternative producer of antioxidant compounds. Microalgae are often considered a mother lode of high value pharmaceutically important metabolites, like carotenoids, polyphenols, fatty acids, phycobiliproteins, vitamins, which are the outcomes of defense strategies of microalgae against stress factors (Chu, 2013). These bioactive compounds have proven antioxidant capability as well as *in vitro* and *in vivo* anticancer property as well. For example, microalgal tetraterpenoids are a good source of antioxidants and also have shown promising antitumor activity in different cell lines (Ferdous and Yusof, 2021). The activity of microalgal antioxidants is commensurate with or sometimes higher than that of plant or animal origin, which makes them a good supply of nutraceuticals for human health (Sansone and Brunet, 2019). Microalgae are getting more attention to exploit in pharmaceutical usage due to having a diverse and wide array of metabolites, accelerated growth rate, ability to grow to disregard the seasonal variation or extremity, not requiring cultivable land and supply of fresh water, and most importantly, not affect food crops (Khan et al., 2018). Microalgae and their metabolites, like astaxanthin, DHA are used popularly as a supplement. *Chlorella* and *Spirulina* are the two most commonly consumed healthy foods in the forms of powder, tablets, or capsules. Currently, *Tetraselmis* is joining the race, which is consumed as an antioxidant supplement. Microalgae-enriched food products are also a good source of nutraceuticals (Koyande et al., 2019). Additionally, seaweeds are also a good source of antioxidant molecules. Among these bioactive, fucoidans, phlorotannin, laminarin, and terpenoids are widely studied for their antioxidant activity (Gupta and Abu-Ghannam, 2011). Moreover, many Asian countries, like China, Indonesia, Japan, Korea, Malaysia, Thailand, and the Philippines, are the leading producers and consumers of edible seaweeds that contain these antioxidants in high amounts (Ferdouse et al., 2018).

However, antioxidant phytochemicals found in these algae have been claimed to exhibit chemo-preventive role in normal cells by suppressing radiation or chemotherapy-induced oxidative stress via activation of the antioxidant defense system in cells, prevention of ROS mediated genomic instability, and inhibition aberrant cell proliferation, metastasis, and angiogenesis. On top of these roles, in combination with chemotherapeutic agents, antioxidants can act as therapeutic agents. They can boost oxidative stress in tumor cells, disable transcription factors, switch on apoptosis-related signaling pathways, and impede signaling pathways involved in cell proliferation (Chikara et al., 2018). Nevertheless, there are still some controversies in the utilization of antioxidants in cancer therapy. This review clarifies reactive species as well as oxidative stress, and their roles in cancer development. Then, the classification and mode of action of antioxidants have been explained briefly. Finally, some well-known microalgal and seaweed antioxidants and their potential roles in cancer therapy are described.

## Reactive Species and Oxidative Stress

Free radicals contain one or more unpaired electrons in their atoms’ outermost shell, which makes them strikingly reactive and more unstable. They are formed in our body naturally as byproducts during biological processes or from exogenous sources and can potentially harm cells. (Shrivastava et al., 2019). Free radicals are related to reactive oxygen species (ROS), reactive nitrogen species (RNS), reactive sulfur species (RSS), reactive carbonyl species (RCS), and reactive selenium species (RSeS) (Sies et al., 2017). These reactive species are continuously formed from endogenous and exogenous sources in our body. Endogenous sources comprise intracellular organelles, like peroxisomes, mitochondria, and extracellular components like inflammatory cells (macrophages, eosinophils, and neutrophils). On the other hand, exogenous sources include high ionizing radiation, environmental toxins (pollution, allergens, toxic metals like cadmium, lead, mercury, iron, arsenic, and pesticides, microorganisms, some drugs, cigarette smoke, alcohol, and dietary xenobiotics (Pizzino et al., 2017).

Among these reactive species, ROS are widely studied. ROS is generated in the cytosol by soluble cell components and cytosolic enzymes, on membranes of mitochondria, in the peroxisomes, in the endoplasmic reticulum, on the plasma membrane of the dysfunctional cells, and in the lysosomes (Di Meo et al., 2016). However, ROS is of two classes; one type consists of radicals with an unpaired electron in their outermost shell (superoxide anion, nitric oxide, hydroperoxyl, and peroxyl radicals, and hydroxyl radical); another class comprises non-radical ROS, and these ROS are without unpaired electron but still has the chemical reactivity, even can be changed to radical ROS, e.g., singlet oxygen, ozone, hydrogen peroxide, and hypochlorous acid (Chahal et al., 2018). In cell signaling, ROS can serve as secondary messengers, playing an essential role in a range of cellular processes by stimulating different signal transduction pathways that involve gene activation or cellular growth (Klaunig and Wang, 2018).

ROS reacting with nitric oxide gives rise to RNS and RSS, with thiols (Corpas and Barroso, 2015; Mut-Salud et al., 2015; Sies et al., 2017). RNS, nitrogen-containing oxidants, consist of nitric oxide (NO•) and nitrogen dioxide radical (NO_2_•), peroxynitrite (HNO_3_^−^), as well as other oxides of nitrogen. Similarly, reactive sulfur species (RSS) are sulfur-containing molecules, which include hydrogen sulfide (H_2_S), thiols (RSH), persulfides (RSSH), polysulfides, S-nitrosothiols (RSNO), hydrogen polysulfides, and sulfenic acids (RSOH), that have essential roles in the regulation of cellular systems (Xu et al., 2019).

**FIGURE 1:**
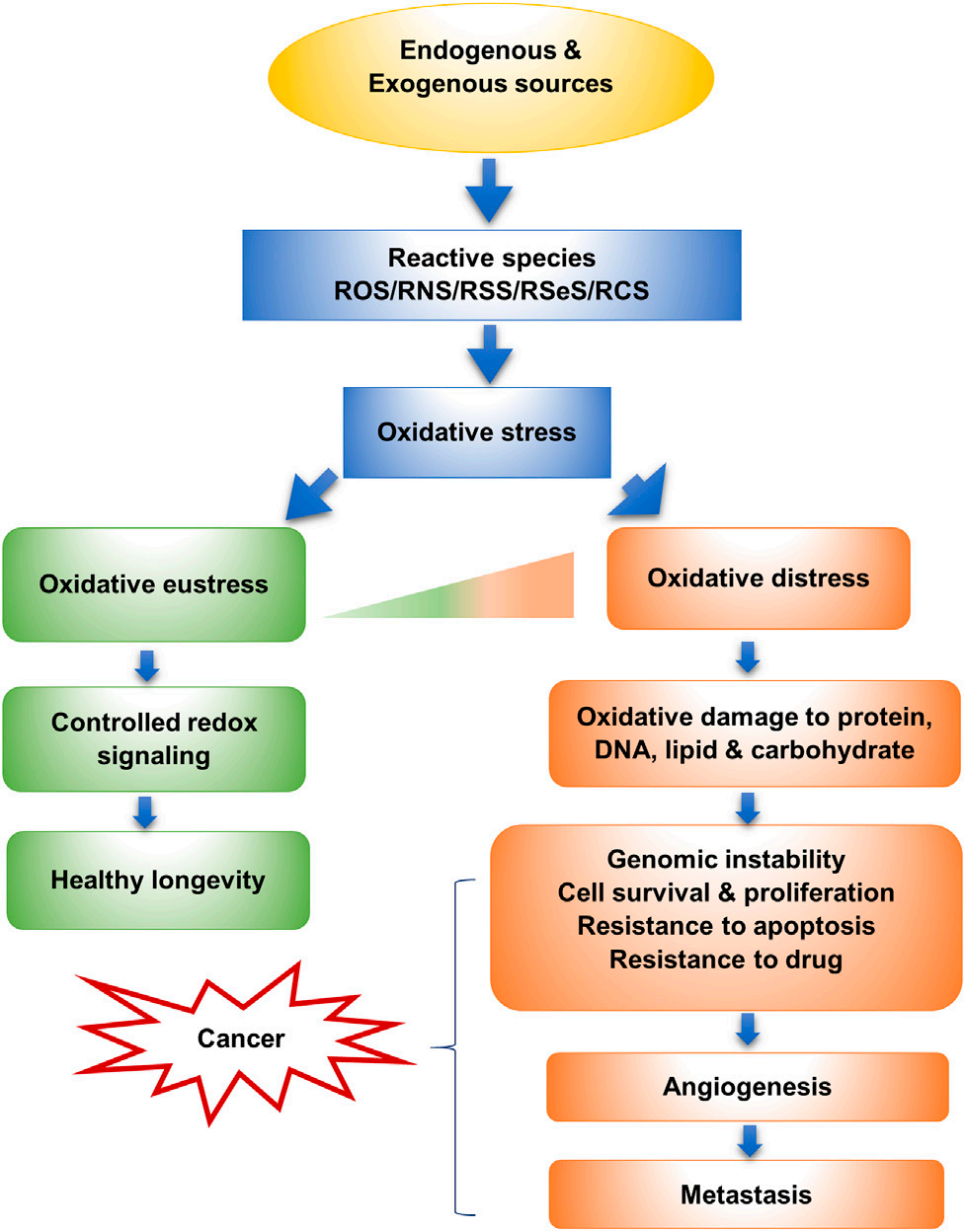
Oxidative stress and its relation to cancer (Sies, 2019).

As a notion in redox biology, the term oxidative stress has been mentioned for the first time in the book entitled “Oxidative Stress” in 1985. Oxidative stress (OS) occurs when there is a disproportion between generation and detoxification of RS by the biological system in cells (Di Meo et al., 2016). According to Helmut Sie, oxidative stress is “an imbalance between oxidants and antioxidants in favor of the oxidants, leading to a disruption of redox signaling and control and/or molecular damage.” Oxidative stress can exert two-sided actions, classified according to intensity, as oxidative eustress and oxidative distress. Low oxidant or reactive species exposure permits addressing particular targets for redox signaling, essential for maintaining normal physiology, which is called oxidative eustress. The basal level of OS augments the defense system through the expression of antioxidant compounds and proteins, yielding health benefits. Contrarily, excessive oxidant or RS challenge leads to disrupted redox signaling, causing deleterious effect, like macromolecular damage in intracellular organelles, inactivation of redox regulatory enzymes, or abnormal cellular proliferation and death, which is termed as oxidative distress (Niki, 2016; Go and Jones, 2017; Sies, 2020) (Figure 1). There are different types of oxidative stress which depend mainly on the generation source, such as nutritional, postprandial, photooxidative, radiation-induced, reductive, and nitroxidative, nitrosative, nitrative oxidative stress (Sies, 2019).

## Effect of Reactive Species and Oxidative Stress on Cancer Cells

OS can play an important role in all phases of the oncogenic process (initiation, promotion, and progression), by activating different transcription factors, including nuclear factor (NF-κB), Nuclear factor erythroid 2-related factor 2 (Nrf2), hypoxia-inducible factor (HIF-1α), activator protein (AP), tumor protein (p53), β-catenin/Wnt signaling pathway, which helps in modulating the expression of immune and inflammatory-related genes and thus triggers carcinogenesis (Saed et al., 2017). Besides, ROS functions bidirectionally in cancer. It can be pro- and antitumorigenic. ROS can contribute to cancer development via a range of cancer signaling pathways, such as MAPK/AP-1/NF-κB, associated with cancer metastasis and angiogenesis. ROS can also trigger inflammation by activating NF-κB, AP-1, HIF-1a, growth factors, inflammatory cytokines, and chemokine. Conversely, elevated ROS level promotes oxidative stress-induced cancer cell death by triggering antitumorigenic signaling (Reczek and Chandel, 2017; Kashyap et al., 2019). Cancer cells always need to keep an elevated ROS level allowing the pro-tumorigenic cell signaling without inducing cell death. Moreover, the ROS scavenging mechanism is stimulated by tumor cells to maintain ROS levels below the cytotoxic level (Ilghami et al., 2020).

### Role of Reactive Oxygen Species in Cell Proliferation and Survival

An increase in ROS has been implicated in enhanced cell growth, proliferation, survival and in the progression of carcinogenesis by regulating mitogen activated-protein kinase, protein kinase D (PKD) signaling pathways, transcription factors such as AP, NF-κB, HIF-1α and also through the negative regulation of phosphatases and protein tyrosine phosphatase 1B (PTP1B), epigenetic alterations in transcription factors and tumor suppressors, Nrf2 and p53, as well as by down-regulating the expression of E-cadherin tumor suppressor (Galadari et al., 2017; Moloney and Cotter, 2018).

### Role of Reactive Oxygen Species in Genetic Instability

ROS often act as mediators of DNA damage. When ROS accumulate cells through its overproduction, they are often associated with DNA interaction, producing ROS-interacting modification, such as inter-and intra-strand bindings or creating DNA-protein crosslinks, yielding altered gene expression. ROS cause DNA damage through oxidizing nucleoside bases and form DNA lesions, such as the formation of 8-oxo guanine, that generate DNA double-strand breaks (DSBs), if unrepaired. ROS accumulation creates mitochondrial DNA lesions, strand breaks, and finally, degradation. In addition, increased ROS through the activation of oncogenes influences the replication stress. ROS can oxidize dNTPs that can modify polymerase activity, breakdown of replication forks, and the formation of DSBs, which all together lead to genomic instability. Moreover, ROS induce activation of proteins associated with cell cycle checkpoint, leading to cell cycle arrest. Above all, these alterations of chromosomes give rise to genetic instability and ultimately lead to carcinogenesis (De Sá Junior et al., 2017; Srinivas et al., 2019).

### Role of Reactive Oxygen Species in Cell Death

Increased ROS engender cell cycle arrest, senescence, and apoptosis. Elevated intracellular ROS production promotes apoptosis via extrinsic or intrinsic pathways. Moreover, ROS trigger apoptosis by inactivating or enhancing the ubiquitination of anti-apoptotic protein, Bcl-2, and by reducing the levels of apoptosis regulator, Bax, and Bad. On the other hand, ROS can kill cancer cells through autophagy, an effective defense against OS damage. ROS cause inactivation of autophagy-related genes and can inhibit the negative regulator of autophagy (TORC1). ROS generated in the mitochondrial electron transport chain or by NADPH oxidases (NOXs), enhance necroptosis. Furthermore, tumor suppressor protein p53 causes cell death through ferroptosis (depends on intracellular iron) which is induced by increased ROS level (Perillo et al., 2020).

### Role of Reactive Oxygen Species in Angiogenesis and Metastasis

In metastasis, tumor cells are circulated from the primary site to other places in the body via blood and lymph. ROS can cause metastasis by inducing hypoxia-mediated MMPs (matrix metalloproteinases) and cathepsin expression. Increased ROS level may activate the MMP enzymes with the stimulation or modulation of a myriad number of tumor progression pathways or metastasis signaling pathways, respectively. Tumor migration can be caused by ROS providing that they are produced by activated growth factor receptors and integrin assembly and with the modulation of signaling kinases. ROS mediate FAK (cell motility controlling protein) activation, leading to cellular invasion. Moreover, ROS can activate the actin-binding protein, cofilin, and thus, help in cell migration.

However, metastasis can be induced by ROS by other mechanisms also, like proteolytic degradation of glycosaminoglycan (GAG) and other ECM components. An increased level of ROS can stabilize HIFα by impeding prolyl hydroxylases (PHDs) and, thus, VEGF (primary pro-angiogenic factor) activation, ultimately rendering angiogenesis and tumor progression (Galadari et al., 2017; Kashyap et al., 2019).

### Role of Reactive Oxygen Species in Chemoresistance

Chemoresistance is a primary cause of treatment ineffectiveness in cancer. P-glycoprotein (a transporter protein) is a multidrug resistance protein that involves the removal or efflux of several anticancer drugs from cancer cells. ROS can upregulate this protein, leading to chemoresistance and inhibiting cell death (Galadari et al., 2017).

## Antioxidants

Antioxidant was first defined by Halliwell et al., in 1989 as “any substance that, present in low concentrations compared to oxidizable substrates (carbohydrates, lipids, proteins or nucleic acids), significantly delays or inhibits the oxidation of the mentioned substrates” (Halliwell et al., 1992). The term ‘Antioxidant’ denotes that antioxidants are molecules that work against the activity of oxidants. Antioxidants can be defined as, chemicals that can inhibit or quench free radicals, that are formed as natural byproducts in the body during the biological process, and thus retarding oxidative damage (Chahal et al., 2018; Khurana et al., 2018).

Antioxidants, which are produced in our body through the metabolic process, are called endogenous antioxidants. Antioxidants can also be incorporated exogenously through foods and dietary supplements, which are called exogenous antioxidants. Besides, there is also another group of antioxidants that can be produced synthetically, which are widely used in the food industry (Mut-Salud et al., 2015).

## Classification of Antioxidants

**FIGURE 2:**
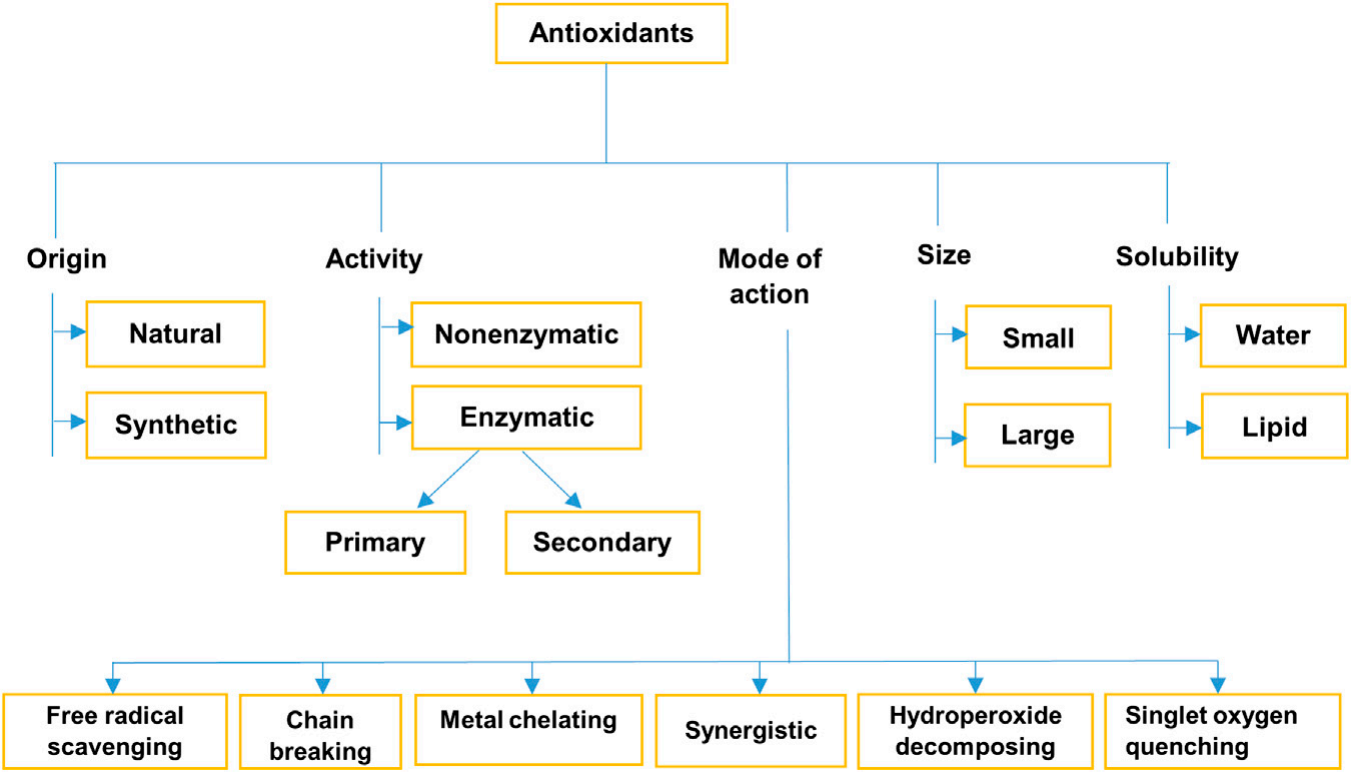
Classification of antioxidants (Nimse and Pal, 2015; Anwar et al., 2018; Chahal et al., 2018; Khurana et al., 2018; Azat Aziz et al., 2019).

Antioxidants can be classified based on their origin, activity, size, solubility, and mode of action (Figure 2).

## Antioxidant Defense System in Cell

Antioxidants give protection to the cells through three lines of defense. The first line of defense includes antioxidants hindering the formation of new free radicals. Enzymatic antioxidants such as SOD, CAT, GPx, and reduced glutathione; metal-binding proteins (ferritin and ceruloplasmin) and antioxidant minerals such as selenium, copper, and zinc. The second line comprises antioxidants, which are involved in scavenging free radicals, and thus preventing OS. Endogenous and exogenous antioxidants such as glutathione, albumin, CoQ, flavonoids, carotenoids, uric acid, and vitamins (A, C, and E) are involved in this group. Finally, different enzymatic antioxidants are the main player in the third line of defense, that repair the damaged DNA, intracellular protein, and other biomolecules. For example, DNA repair enzymes, proteases, peptidase, lipases, transferases, etc. (Surai et al., 2003; Mut-Salud et al., 2015).

## Effect of Antioxidants in Cancer Therapies

Cancer is a term used for a cluster of analogous diseases that causes cells anywhere in the body commence to divide out of control and start proliferating in the surrounding or even distant tissues. It is the second-highest cause of mortality globally and accounts for approximately 9.6 million deaths in 2018 (WHO, 2020). Depending on the type of cancer and the malignancy, there is a range of cancer treatments, such as surgery, chemo-, radiation-, immuno-, targeted- and hormone therapy, stem cell transplant, or a combination of these therapies. Among them, chemotherapy remains the treatment of choice, integrated with surgery or other therapies. Commonly used chemotherapy drugs are the alkylating agents, anthracyclines (doxorubicin, daunorubicin, epirubicin, idarubicin, aclarubicin, and pirarubicin), epipodophyllotoxines, platinum-based drugs (cisplatin, carboplatin, and oxaliplatin), camptothecins, vinca alkaloids, taxanes, and antimetabolites, which are used for the treatment of a variety of cancers, such as breast, liver, ovarian, testicular, bladder, head and neck, lung cancer (He et al., 2018; Moiseeva, 2019; Ilghami et al., 2020). These drugs can cause more than 40 specific side effects and are broadly categorized into seven types, namely cardiotoxicity, hepatotoxicity, nephrotoxicity, ototoxicity, neurotoxicity, hematological toxicity, and gastrointestinal toxicity. (Oun et al., 2018). On the other hand, radiation therapy uses ionizing radiation to kill cells, by generating ROS, other organic radicals, and lipid peroxidation. Therefore, radiation induces an increase of free radicals which damage DNA and ultimately leads to cell death. This elevated ROS can affect the cellular antioxidant status as well (Mut-Salud et al., 2015; Ko and Formenti, 2019).

The goal of cancer treatment should be to kill cancer cells successfully and be attenuating therapy-induced genotoxicity in normal tissues and detoxifying harmful effects after treatment should be an additional goal of cancer treatment (Vilimanovich and Jevremovic, 2019). Therefore, antioxidant supplementation is often recommended to neutralize the effects of these chemotherapy drugs.

The usage of antioxidant supplements during cancer therapy can reduce oxidative damage in the surrounding healthy tissues, reduce side effects, and boost overall patient health and survival rate. (Calvani and Favre, 2019). These supplements can decrease cell growth, inhibit cell proliferation, and induce apoptosis in tumor cells. However, it has been estimated that 20–85% of cancer patients use antioxidant supplements, where the majority of consumers are breast cancer survivors. Also, patients with prostate, colorectal, and lung cancers prefer to take these supplements. When combined with certain types of chemotherapy, these nutraceuticals become more beneficial in treating cancer (Calvani et al., 2020).

## Potential Microalgal Antioxidants for Use in Cancer Therapies

**FIGURE 3:**
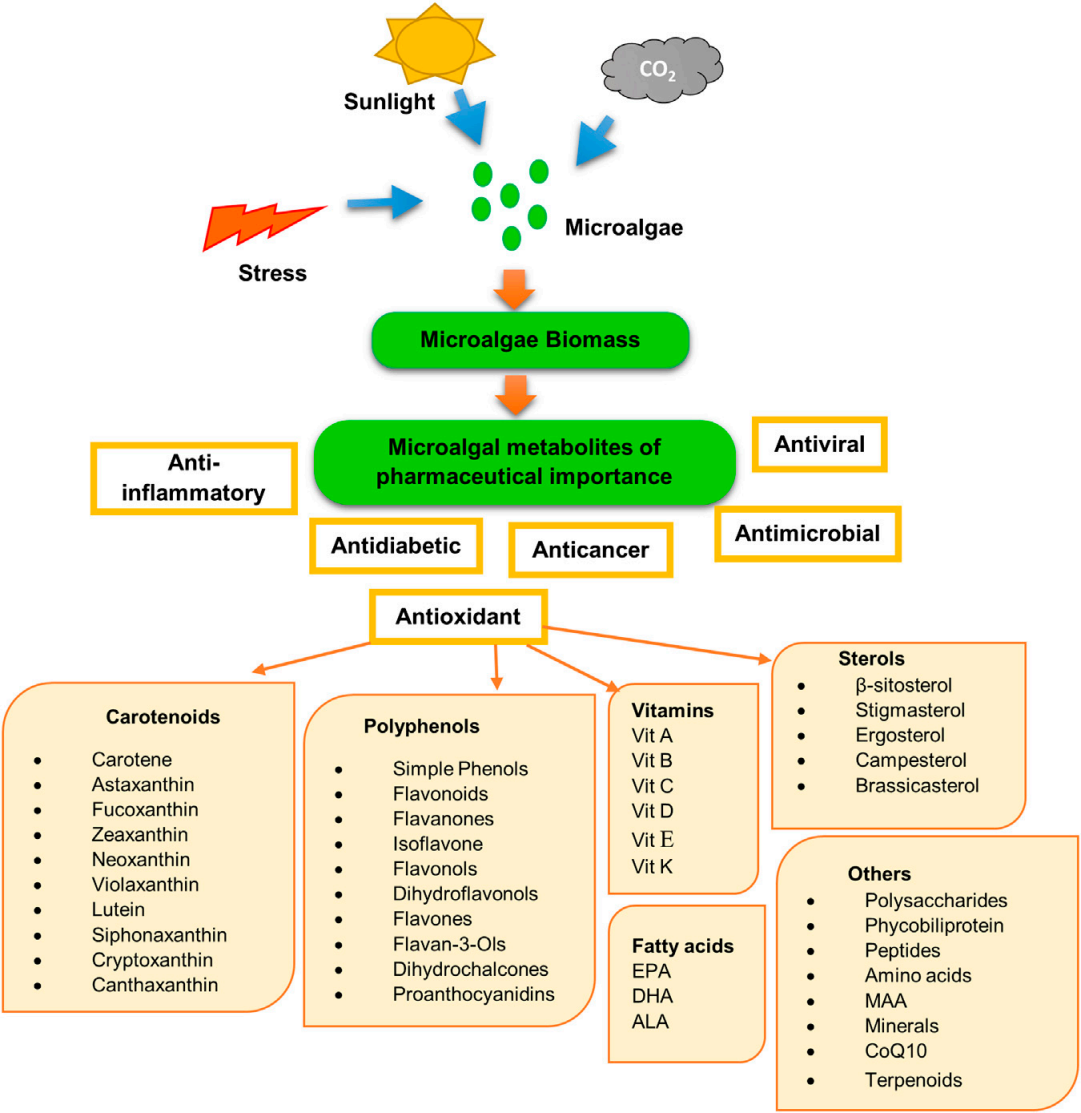
Production of antioxidants from microalgae.

Microalgal antioxidants are primarily composed of carotenoids, phenolics, flavonoids, polyunsaturated fatty acids, vitamins, sulfated polysaccharides, sterols, minerals, amino acids, phycobiliproteins as well as some other compounds like MAA, sulfolipids, Coenzyme Q, and peptides (Figure 3). From blue-green algae, antioxidant components like scytonemin, C-phycocyanin are also known as strong cytotoxic agents (Abd El-Hack et al., 2019). These phytochemicals have anti-cancerous properties as well (Table 1).

**TABLE 1:** List of some antioxidants found in microalgae that showed *in vitro* antitumor activities.

Algae	Antioxidants	Targeted cell line	Active concentration	Mode of action	References
*Dunaliella salina*	β-carotene	Human prostate cancer cell line (PC-3)	Inhibition rate: 79% at 50 µM	Apoptosis associated with mitochondrial dysfunction and DNA fragmentation	(Jayappriyan et al., 2013)
*Dunaliella tertiolecta*	Violaxanthin	Human breast cancer cell line (MCF-7)	^a^IC_50_: 56.1 µg/ml	Apoptosis	(Pasquet et al., 2011)
*Porphyridium purpureum*	Zeaxanthin	Human melanoma cell line (A2058)	IC_50_: 40 µM	Gene mutation; activation of pro-apoptotic factors; cell cycle arrest; caspase activation; inhibition of NF-κB	(Juin et al., 2018)
*Chaetoceros calcitrans*	Fucoxanthin	Human liver cancer cell line (HepG2)	IC_50_: 18.89 μg/ml	Gene modulation of cell signaling, apoptosis, and oxidative stress	(Foo et al., 2018)
*Chlorella ellipsoidea*	Carotenoids (mainly violaxanthin)	Human colon cancer cells (HCT116)	IC_50_: 40.73 ± 3.71 μg/ml	Apoptosis	(Cha et al., 2008)
*Chlorella vulgaris*	Carotenoids (mainly lutein)	Human colon cancer cells (HCT116)	IC_50_: 40.31 ± 4.43 μg/ml	Apoptosis	(Cha et al., 2008)
*Codium fragile*	Siphonaxanthin	Human leukemia cells (HL-60)	Inhibition rate: 95% at 20 µM	Activation of caspase-3; up-regulation of GADD45a and DR5, downregulation of Bcl-2	(Ganesan et al., 2011)
*Cyanophora paradoxa*	β-Cryptoxanthin	Malignant	Inhibition rate: 93.0 ± 0.1% at 100 μg/ml	Apoptosis	(Baudelet et al., 2013)
		Melanoma cells (A-2058)			
*Haematococcus pluvialis*	Astaxanthin	Human hepatoma cancer cell line (HepG2)	Inhibition rate: 58.55% at 25 μg/ml	Depletion of glutathione; DNA fragmentation; cell cycle arrest at G0/G1 phase	(Nagaraj et al., 2012)
*Nannochloropsis oculata*	Sterols	Human promyelocytic leukemia cell line (HL-60)	IC_50_: 23.58 ± 0.09 μg/ml	Apoptosis	(Sanjeewa et al., 2016)
*Nannochloropsis salina*	PUFA	Human breast cancer cell line (MCF-7)	IC_50_: 0.45 μg/ml	-	(Sayegh et al., 2016)
*Porphyridium cruentum*	Sulfolipids	Human adenocarcinoma cells	IC_50_: 20–46 μg/ml	Cell cycle arrest; inhibition of DNA polymerase	(Bergea et al., 2002)
*Phaeodactylum tricornutum*	Sulfated polysaccharides	HepG2	Inhibition rate: 60.37% at 250 μg/ml	Apoptosis	(Yang et al., 2019)
*Tribonema* sp	Sulfated polysaccharides	HepG2	Inhibition rate: 66.8% at 250 μg/ml	Apoptosis	(Chen et al., 2019)
*Chlorella zofingiensis*	Exopolysaccharides	Human colon cancer cell lines (HCT8)	IC_50_: 1.7 mg/ml	-	(Zhang et al., 2019)
*Spirulina platensis*	C-Phycocyanin	Human breast cancer cell lines (MDA-MB-231)	IC_50_ : 189.4 μg/ml	Cell cycle arrest at G0/G1 phase; decreased levels of cyclin D1 and CDK-2 and increased levels of p21 and p27; down regulation of cyclooxygenase-2; activation of MAPK signaling pathways	(Jiang et al., 2018)

^a^ IC_50_: the concentration needed for 50% inhibition.

### Vitamins

#### Vitamin A

Vit A comprises retinol and its derivatives (retinoids). It is a collective term for many analogous compounds that can be classified into two groups based on the source. Vit A is derived from animal-based foods, such as beef liver, eggs, cod liver oil, butter, and yellow pigmented fruits, vegetables, and fortified grains, which are called preformed vitamin A. On the other hand, provitamin A (α- and β-carotene, β-cryptoxanthin) are available in colored fruits and vegetables, like in tomato, carrots, leafy greens, yams, and vegetable oils. This retinol is changed into retinoic acid and retinoids upon entering into the body (Fritz et al., 2011; Kim J. et al., 2019). Interestingly, some microalgae species contain Vit A which is much higher in amount compared to some fruits. For instance, *Tetraselmis suecica* contains Vit A (493,750 I.U./kg dry weight) in a higher amount than orange (14,728 I.U./kg dry weight). *Isochrysis galbana*, *Dunaliella tertiolecta*, *Chlorella stigrnatophora*, *Chaetoceros calcitrans* and *Skeletonema costatum* are also a rich source of Vit A and provitamin in comparison with other foods like cod liver oil, beef liver or parsley (Fabregas and Herrero, 1990; De Roeck-Holtzhauer et al., 1991). Cyanobacteria *Aphanizomenon flos-aquae* and *Spirulina* spp. are another reservoir of provitamin A (Kay and Barton, 1991). Chronopoulou et al., has reported that extraction of vit A from *Tetradesmus Obliquus* is in the highest amount through supercritical fluid extraction method (Chronopoulou et al., 2019).

Higher intake of dietary Vit A can remarkably decrease the ovarian, lung, gastric, pancreatic, and cervical carcinoma risk (Sanusi, 2019; Wang and He, 2020). Retinoic acid activates the extracellular-signal-regulated kinase (ERK) pathway and thus, promotes angiogenesis and metastasis in lung cancer. Retinoids in combination with chemotherapeutic drugs and other antioxidants inhibit cancer cell proliferation and thus increase the life span of cancer patients (Tripathi et al., 2019). Besides, natural and synthetic retinoids can prevent colorectal cancer progression (Abdel-Samad et al., 2019). Furthermore, retinol has a protective capacity against digestive cancers (Xie et al., 2019). Additionally, an increased dietary supplement of Vit A and β-carotene can improve hepatocellular carcinoma prognosis with an increased survival rate (Zhang et al., 2020).

#### Vitamin C

Vit C can be obtained naturally in a variety of fruits, vegetables like green chili, thyme, parsley, guavas, black current, kiwis, lemon, and algae. It is commonly called ascorbic acid and is aqueous soluble (Padayatty and Levine, 2016). It is often considered a well-tolerated micronutrient. Vit C containing eleven microalgae species from different classes have been reported where *Chaetoceros muelleri*, *Skeletonema costatum*, *Nannochloropsis oculata*, and *Nannochloris atomus* showed higher amount of Vit C than others (Brown et al., 1997). Vitamin C is also commonly found in *Spirulina* spp.*, Chlorella* spp., *T. suecica*, *I. galbana*, *D. tertiolecta*, *Aph. flos-aquae*, *Pavlova lutheri*, and *Rhodomonas salina* (Fabregas and Herrero, 1990; Kay and Barton, 1991; Brown et al., 1997).

However, Vit C can highly sensitize tumor cells compared to normal cells. Vit C acts as a prodrug by generating ascorbate radicals and H_2_O_2,_ causing oxidative stress and ultimately kill cancer cells, which can be attained by the intravenous injection of Vit C. On the other hand, cancer cells can be damaged through epigenetic regulation, like DNA and histone demethylation, and reestablishing 5-hydroxymethylcytosine with oral administration of Vit C supplement. Moreover, Vit C supplementation can prevent tumor metastasis by collagen cross-linking, suppress cancer progression by HIF-1a degradation (Mustafi and Wang, 2019). It has been reported that higher doses of ascorbic acid alone or combinedly with conventional cancer drugs significantly impede cancer growth, but it should be administered intravenously. Oral administration of ascorbate causes only a moderate increase in its plasma concentration (Blaszczak et al., 2019).

In contrast, another study revealed that intravenous administration of Vit C of a lower dose with longer administration was better in treating cancer, though a high dose is still safe (Mikirova et al., 2019). Vit C can modulate infiltration of the tumor microenvironment by stimulating immune cells and delay cancer growth in breast, colorectal, melanoma, and pancreatic murine tumors (Magrì et al., 2020). Importantly, Vit C can kill cancer cells selectively and its activity depends on factors like the type of cancer and signaling pathways involved in the tumor development. In cancer stem cells, it can enter through sodium-dependent Vit C transporter 2 (SVCT2) and alter JHDM and TET protein. Besides it can enter via glucose transporters (GLUTs) and modify ROS, causing mitochondrial dysfunction and finally triggers Vit C-induced cell death (Satheesh et al., 2020). Vit C supplementation shows a protective effect in modulating inflammatory regulators in the case of esophageal adenocarcinoma (Abdel-Latif et al., 2019).

#### Vitamin D

Vit D is also known as the “sunshine” vitamin since it can be gained through exposure to sunlight. Besides, this fat-soluble vitamin is available in foods like fishes rich in fat, egg yolk, dairy products like cheese, cod liver oil, beef liver, and mushrooms. Surprisingly, *P. lutheri*, *T. suecica*, *S. costatum*, and *I. galbana* can produce Vit D in a higher amount in comparison with cod liver oil, oyster, mushroom, egg, and liver (De Roeck-Holtzhauer et al., 1991). It has been reported that Vit D_3_ is found in the highest amount in UVB exposed *Nannochloropsis oceanica* (Ljubic et al., 2020).

In the liver, Vit D is metabolized into 25(OH)D (25-hydroxyvitamin D), which is a biomarker for Vit D status assessment (Marian, 2017). Studies showed that daily supplementation of Vit D is effective in improving relapse-free survival among digestive tract cancer patients and also in early-stage lung adenocarcinoma, with low bioavailable 25(OH)D levels (Akiba et al., 2018; Urashima et al., 2020). Besides, Vit D supplementation can reduce cancer-related mortality (Keum et al., 2019). Additionally, in the kidney, it is conceived that 25(OH)D can be converted to calcitriol by 1-alpha hydroxylase that can attach to Vit D specific receptors and has a significant effect on gene expression, and thus control cancer cell survival (Chatterjee et al., 2019).

#### Vitamin E

Vit E, a lipid-soluble vitamin, is mainly found in nuts, seeds, vegetables, plant oils. Marine microalgae are an excellent source of Vit E and contain a larger amount of Vit D than other plant and animal sources. Studies showed that *C. stigmatophora*, *C. calcitrans*, *P. lutheri*, *T. suecica*, *S. costatum*, *I. galbana,* and *D. tertiolecta* possess ample amount of Vit D than olive oil, corn, bean, carrot, wheat or liver (Fabregas and Herrero, 1990; De Roeck-Holtzhauer et al., 1991). This Vit E can be classified into eight isoforms, namely α, β, δ, γ-tocopherol, and -tocotrienol (Peh et al., 2016). *T. obliquus* contains α and γ-tocopherol (Chronopoulou et al., 2019). *Chlorella* spp., *Spirulina* spp. and *Aph. flos-aquae* also have a significant level of Vit E (Kay and Barton, 1991; Kim et al., 2001).

Intake of vitamin E supplementation up to upper tolerable intake level (UL) of 300–1,000 mg/day is considered safe and effective in the reduction of mortality (Köpcke, 2019). Vitamin E supplementation has significant neuroprotective properties against cisplatin-induced ototoxicity (Villani et al., 2016) and also in cisplatin-induced nephrotoxicity, where a significant reduction in the serum levels of renal injury biomarker (NGAL) has been observed (Ashrafi et al., 2020). It has been reported that intake of high Vit E supplementation reduces total cancer and gastrointestinal cancer risk among patients with high selenium levels (Wang et al., 2019). Tocotrienols can selectively suppress cancer cells without harming the normal cells, where γ and δ tocotrienols have the highest anti-cancer activity. They can exert anti-cancer activity by inhibiting cell proliferation, arresting cell cycle, inhibition of angiogenesis by downregulation various growth factors, metastasis and inducing cell death (apoptosis, autophagy, and paraptosis) through different mechanisms that involve death receptor, caspase 9 activation, or Bax/Bcl ratio (Abraham et al., 2019; Constantinou et al., 2020). Besides, Vit E consumption reduces the risk of bladder cancer (Lin et al., 2019).

#### Vitamin K

Vitamin K belongs to lipid-soluble vitamin, also known as ‘Koagulations vitamin,’ which is divided into two classes Vit K1 and K2, along with synthetic derivatives K3–K5. Vit K1 and K2 are also called phylloquinone and menaquinone, respectively, which are found in leafy vegetables, cheese, and curd (Kurosu and Begari, 2010). Vit K is also available in *T. suecica*, *I. galbana*, *S. costatum*, *P. lutheri*, *Chlorella ellipsoidea*, and *T. obliquus* where the level is significantly higher than milk, egg, or vegetables like spinach, cabbage (De Roeck-Holtzhauer et al., 1991; Kim et al., 2001; Chronopoulou et al., 2019).

Vit K and derivatives have been reported to exhibit anticancer property against cancer in the lung, liver, breast, prostate, blood, colon, and bladder. It can destroy cancer cells through several mechanisms, such as by increasing oxidative stress, by inducing apoptosis through the upregulation of Fas/FasL, NF-kB, p53, downregulating Bcl-2/Bcl-xl, Bax/Bak, and also through caspase-3 activation pathway, by inhibiting cell cycle through the inhibition of CDK-1 checkpoint and activation of CDK-1 inhibitors, p21. It can also induce autophagic death in different cancer cells (Dasari et al., 2017). Along with autophagy, Vit K2 can cause non-apoptotic cell death in breast cancer cell lines (Miyazawa et al., 2020). In combination with sorafenib, Vit K1 can cause apoptosis in hepatocellular carcinoma cells *in vivo* and *in vitro* through the activation of caspase pathways (Wei et al., 2010). In prostate cancer, Vit K2 has been reported to hinder metastasis and inducing apoptotic cell death (Vinjamuri et al., 2019).

### Polyphenols

Microalgae is a rich source of polyphenolic compounds that mainly consist of simple phenols, flavonoids, flavanones, isoflavone, flavonols, dihydroflavonols, flavones, flavan-3-ols, dihydrochalcones, proanthocyanidins. Among them, Flavones (Apigenin) and isoflavone (Genistein) have been reported to be found in *P. tricornutum*, *Diacronema lutheri*, *P. purpureum*, *H. pluvialis*, *T. suecica,* and *C. vulgaris,* while *D. lutheri* and *H. pluvialis* contain the most diverse classes of flavonoids (Goiris et al., 2014). In a study, Bulut et al., (2019), has assumed that flavonol (quercetin) from *Scenedesmus* sp., is one of the major contributors to its antioxidant property (Bulut et al., 2019). On the other hand, marine microalgae *P. tricornutum*, isolated from the Moroccan sea, produce protocatechuic acid which is considered to have antioxidant activity (Haoujar et al., 2019).

*Euglena cantabrica* having a high amount of phenolics (gallic acid and protocatechuic acid) shows the most effective radical scavenging activity which was even more than the conventional antioxidants (Jerez-Martel et al., 2017). Phenolic acids from *Spirulina maxima* displayed better radical scavenging activity and protection against microsomal lipid-peroxidation in the liver than commercial antioxidants (Abd El-Baky et al., 2009). Phenolic compounds are responsible for antioxidant activity tested for a myriad of microalgae, for instance, *Nannochloropsis* sp., *Spirulina* sp., *D. salina*, *Navicula clavata*, *Chlorella* sp., *Tetraselmis* sp*., Porphyridium cruentum*, *P. tricornutum*, *Neochloris oleoabundans*, *C. calcitrans*, *Botryococcus braunii* (Goiris et al., 2012; Hemalatha et al., 2013; Choochote et al., 2014; Zainoddin et al., 2018).

Along with antioxidant activity, these polyphenolic compounds exhibit anticarcinogenic activity as well. Jayshree et al., (2016), found that flavonoids, isolated from *C. vulgaris* as well as *Chlamydomonas reinhardtii*, were cytotoxic to breast cancer cells (Jayshree et al., 2016). Similarly, flavonoids in *C. vulgaris* extract can hinder proliferation in lung carcinoma (Wang et al., 2010). *Spirulina maxima* produce phenolic compounds that may stop proliferation and induce apoptosis in liver cancer cells (Wu et al., 2005). Likewise, phenolic compounds from *C. vulgaris* and *I. galbana* might be responsible for the anticancer activity against human liver cancer (Custódio et al., 2014; Raikar et al., 2018).

Polyphenols like quercetin, genistein, ellagic acid have significant anticancer properties. Genistein has displayed anticancer effects against breast, colon, lung, thyroid, gastric, and prostate cancers by modulating a variety of molecular targets, such as apoptotic markers caspases, Bcl and Bax, nuclear factor-κB, an inhibitor of NF-κB, mitogen-activated protein kinase (MAPK), phosphoinositide 3-kinase/Akt (PI3K/Akt), extracellular signal-regulated kinase 1/2 (ERK 1/2), and Wingless and integration 1/β-catenin (Wnt/ß-catenin) signaling pathway (Tuli et al., 2019). It can show pro-apoptotic, anti-proliferative, and anti-metastatic activities *in vitro* on PC3 prostate cancer cells through triggering apoptosis by activating caspase-3 related pathways, by reducing cell survival via inhibition of p38MAPK at both gene expression and protein levels, and by inhibiting metastasis through the blockage of MMP2 activity (Shafiee et al., 2020). Genistein is documented as clinically safe and effective when combined with standard fluoropyrimidine and platinum-based drug, oxaliplatin, with or without Bevacizumab, in the treatment of metastatic colon cancer (Pintova et al., 2019). Quercetin exerts its anti-cancer effects on different cancer cells through the regulation of PI3K/Akt/mTOR, Wnt-catenin, and MAPK/ERK1/2 pathways. It can induce tumor cell death by modulating the apoptotic pathway, enhancing the expression of pro-apoptotic proteins (Bax, Bad) as well as decreasing the expression level of anti-apoptotic proteins (Bcl, Mcl), and also affect the expression of TRAILR, FAS, TNFR1. Moreover, it hinders metastasis by reducing VEGF secretion, repressing the expression of the downstream regulatory factor AKT and MMP levels, and by inhibiting EMT progression. Furthermore, quercetin promotes protective autophagy in cancer cells by forming autophagic vacuoles and acidic vesicular organelles (AVOs), activating autophagic gens, and inhibiting Akt-mTOR signaling and stabilizing HIF-α expression (Reyes-Farias and Carrasco-Pozo, 2019; Tang et al., 2020). However, coadministration of sorafenib (0.1 µM) and quercetin 25 µM for 1 day has been exhibited a significant reduction in the cell proliferation rate and inhibition in cell adhesion and migration properties (Celano et al., 2020).

Another important phenolic compound in microalgae is ellagic acid (EA). EA can effectively reduce cisplatin (CP) induced nephrotoxicity and gonadotoxicity, by reducing peroxidative damage to tissue, when given together with CP to murine colon cancer model (Goyal et al., 2019). Moreover, EA in combination with doxorubicin and cisplatin can strongly hinder cell proliferation and engender mitochondria-mediated cell death in hepatocellular carcinoma cells *in vitro* and reduce side effects significantly (Zhong et al., 2019). In the multidrug-resistant glioma cells, EA combined with bevacizumab may show both inhibitory and suppressive role in bevacizumab-induced DNA repair, when treated for an extended period (Çetin et al., 2019).

### Carotenoids

#### β-Carotene

β-Carotene (BC) is abundantly found in the human diet and popularly used as a food additive and coloring agent in the food industry (Bogacz-Radomska and Harasym, 2018). Microalgae *Dunaliella salina* possesses a copious amount of BC and is considered the richest source among other microalgae. BC from *Dunaliella salina* has been reported to kill human prostate cancer cells through apoptosis (Jayappriyan et al., 2013). Moreover, BC can be found readily in green microalgae *Chlorella vulgaris*, *Asterarcys quadricellulare,* and in cyanobacteria *Spirulina* sp. (Seshadri et al., 1991; Damergi et al., 2017; Singh et al., 2019).

BC suppresses the proliferation and self-renewal capacity of colon cancer stem cells (CSCs) through epigenetic modulation, involving expression of miRNAs and miRNA-mediated histone acetylation, and global DNA methylation (Kim D. et al., 2019). Though its negative relationship to lung cancer is widely studied, it can reduce lung cancer when combined with vitamin A (Yu et al., 2015). It has been documented that oral administration of beta-carotene-loaded solid lipid nanoparticles (BC-SLNs) enhances the bioavailability of BC and also the safety as well as the efficacy of BC. It sustains the release of BC from the lipid core and prolongs circulation time in the body (Jain et al., 2019). Besides, in methotrexate (MTX) therapy, BC loaded nanoparticles of zein (βC-NPs) significantly improve cellular uptake, reduces MTX-induced liver and kidney toxicity, and display elevated biopharmaceutical performance of BC orally (Jain et al., 2019).

#### Lutein

Lutein is a carotenoid with a yellow-orange hue that is an important ingredient in the food, feed, and pharmaceutical industries. It is available in fruits, vegetables, and flowers, especially in marigold which is considered as a primary source (Becerra et al., 2020). Surprisingly, microalgae can produce up to six times higher lutein content compared to marigold and thus, is claimed to be a better alternative of lutein production (Lin et al., 2015). Lutein is produced at a higher amount in *Chlorella protothecoides*, *C. sorokiniana*, *C. vulgaris*, *H. pluvialis*, *Parachlorella* sp*.*, *Muriellopsis* sp. and *Scenedesmus obliquus* (Li et al., 2001b; Shi et al., 2002; Blanco et al., 2007; Chan et al., 2013; Chen et al., 2016; Di Sanzo et al., 2018; Heo et al., 2018). Lutein from *Botryococcus braunii* has been reported to exhibit both *in vitro* and *in vivo* antioxidant activity (Rao et al., 2006).

Lutein augments the effect of the antiproliferation and apoptosis capacity of chemotherapy drugs and also can inhibit cell cycle progression, alone or combinedly with chemotherapy drugs, in the prostate cancer cell line. Moreover, lutein downregulates biomarker genes related to growth and survival in prostate cancer (Rafi et al., 2015). In another study, lutein has been shown anti-breast cancer activity by generating intracellular ROS level and also by inducing apoptotic cell death via downregulation of Bcl2 genes with the upregulation of pro-apoptotic genes and by enhancement of p53 signaling pathway. At the same time, lutein augments the anticancer activity of taxanes when administered combinedly in breast cancer cell lines (Gong et al., 2018). A similar result has been reported by Luan et al., (2018), where lutein plus doxorubicin hinders the growth of sarcoma cells, induces apoptosis, and also shows *in vivo* anti-tumor activity in a mouse model (Luan et al., 2018). Lutein also displays anti-proliferation activity in breast cancer cells by triggering the NrF2/ARE pathway and inactivating the NF-κB signaling pathway (Chang et al., 2018).

#### Astaxanthin

Astaxanthin (ATX), a red lipid-soluble xanthophyll carotenoid, is mostly available in microorganisms and has an important role in aquaculture, food, and pharmaceutical industries (Ambati et al., 2014). *Haematococcus pluvialis* is considered the finest production source of ATX industrially (Shah et al., 2016). This ATX from *H. pluvialis* hinders the oxidative stress inside the cells (Régnier et al., 2015). ATX is also obtained from other microalgae like *C. sorokiniana*, *C. zofingiensi*, *Tetraselmis* sp., *Chlorococcum* sp. and *G. sulphuraria* (Li et al., 2001a; Ip and Chen, 2005; Raman and Mohamad, 2012; Graziani et al., 2013).

ATX exhibits anti-proliferation activity against various cancer cells through blocking cell cycle at G0/G1 phase or G2-M phase, epigenetic alterations, or chromatin remodeling. It also induces apoptosis by downregulation of the antiapoptotic proteins while upregulation of the proapoptotic proteins. It also blocks angiogenesis and metastasis to distant tissues (Faraone et al., 2020). On combinatorial treatment with carbendazim, AXT potentiates the anti-proliferative effect of this drug by arresting MCF-7 cells at the G2/M phase (Atalay et al., 2019).

#### Fucoxanthin

Fucoxanthin (FX) is an orange-hued marine carotenoid that is mainly obtained from algae. Fucoxanthin has many health benefits, especially antioxidative and antiproliferative capacity (Muthuirulappan and Francis, 2013). It has exhibited antitumor activity against a range of cancer types, namely osteosarcoma, leukemia, lymphoma, and also against colorectal, breast, prostate, hepatocellular, bladder cancer (Martin, 2015). Antioxidant activity of FX have been reported from *Phaeodactylum tricornutum*, *Odontella aurita*, *I. galbana*, *C. calcitrans*, *D. salina*, *C. gracilis*, *Navicula* sp., *Thalassiosira* sp*.*, *Pavlova lutheri*, *Cylindrotheca closterium* (Rijstenbil, 2003; Xia et al., 2013; Neumann et al., 2019; Peraman and Nachimuthu, 2019). FX from *P. tricornutum* and *C. calcitrans* has been reported to show anticancer activity as well (Foo et al., 2018; Neumann et al., 2019). Furthermore, FX obtained from *Conticribra weissflogii* showed the anti-inflammatory property in the sepsis mouse model (Su et al., 2019). However, FX is also available in *Nitzschia laevis*, *Chaetoceros muelleri*, *Amphora* sp. and *Tisochrysis lutea* (Ishika et al., 2019; Sun et al., 2019; Mohamadnia et al., 2020).

The anticancer mechanism of FX is mainly directed by blocking the cell cycle at the G0/G1 phase with decreased cyclin D and also by apoptotic cell death with DNA degradation, chromatin condensation, or DNA laddering. FX also inhibits metastasis where a decreased level of MMPs has been observed. Besides, these mechanisms involved a myriad of pro-and anti-apoptotic proteins and many signaling pathways like caspase, PI3K/Akt/mTOR, JAK/STAT, MAPK, SAPK/JNK pathways (Kumar et al., 2013).

#### Zeaxanthin

Zeaxanthin (ZX) is a yellow colored carotenoid and also found in orange or yellow colored fruits, vegetables, like corn, tangerine, squash, mango, honeydew, papaya, peach, yellow bell pepper, marigold, egg yolk, and in many microorganisms as well (Sajilata et al., 2008). On the other hand, ZX can be obtained from microalgae like in *Synechocystis* sp., *Dunaliella salina*, *Chlorella saccharophila*, *C. ellipsoidea*, *C. pyrenoidosa*, *Scenedesmus almeriensis*, *S. obliquus*, *Porphyridium aerugineum*, *Microcystis aeruginosa*, and *Spirulina* sp*.* (Lagarde et al., 2000; Chen et al., 2005; Inbaraj et al., 2006; Granado-Lorencio et al., 2009; Koo et al., 2012; Yu et al., 2012; Singh et al., 2013; El-Baz et al., 2019).

ZX from *Nannochloropsis oculata*, *Scenedesmus obliquus*, *Porphyridium aerugineum* has been reported to show the antioxidative property (Cho et al., 2011; Banskota et al., 2019). On the other hand, the anticancer activity of ZX has been reported in *Porphyridium purpureum*, where ZX induced apoptosis in cells of human melanoma through the augmentation of proapoptotic proteins (Bak, Bax) or pro-apoptotic factors (Bim, Bid) and the reduction of antiapoptotic proteins (Bcl-2), as well as through caspase 3 activation and DNA fragmentation. Moreover, ZX from this *P. purpureum* potentiates the efficacy of the chemotherapeutic drug, vemurafenib toward human melanoma (Juin et al., 2018). A similar apoptosis mechanism of ZX in melanoma cells was reported in another study as well (Bi et al., 2013).

#### Canthaxanthin

Canthaxanthin (CTX), a ketocarotenoid, was found in *Cantharellus cinnabarinus* mushroom for the first time and now is gaining interest in the food and feed industry (De Miguel et al., 2001). This antioxidative and antitumorigenic CTX can be found in microalgae also. Microalgal species like *Haematococcus pluvialis*, *Chlorella emersonii*, *C. zofingiensis*. *Coelastrella* sp*.*, *Dactylococcus dissociates*, *Chlorococcum* sp*.* and also in some cyanobacteria like *Nodularia spumigena*, *Aphanizomenon flos-aqua*, *Trichormus variabilis*, *Anabaena* sp. (Ben-Amotz, 1993; Malis et al., 1993; Li et al., 2006; Nobre et al., 2006; Hu et al., 2013; Grama et al., 2014; Janchot et al., 2019; Krajewska et al., 2019).

CTX showed anticancer activity by causing apoptosis in human colon adenocarcinoma as well as in melanoma cells (Palozza et al., 1998). Similarly, CTX from *Aspergillus carbonarius* has been reported to engender apoptosis in human prostate cancer cells (Kumaresan et al., 2008). Dietary intake of CTX has *in vivo* chemopreventive role in oral cancer (Tanaka et al., 1995).

#### Violaxanthin

Violaxanthin (VLX), an orange-hued carotenoid, is obtained mainly from fruits of similar color and also from leafy greens as well as microalgae. VLX has significant antioxidative activity. Yellow-green microalgae *Eustigmatos cf. polyphem* has been reported to produce VLX that has exhibited radical scavenging activity through DPPH and ABTS assays (Wang et al., 2018). Moreover, VLX has antiproliferative activity as well. VLX isolated from *Dunaliella tertiolecta* and *Chlorella ellipsoidea* has been revealed to inhibit breast cancer cells and colon cancer cells, respectively, and also induce apoptosis (Cha et al., 2008; Pasquet et al., 2011).

However, VLX from *Chlorella vulgaris*, *N. oceanica*, *Dunaniella salina*, *Tetraselmis* spp., *Isochrysis galbana*, *Pavlova lutheri*, *P. salina,* and *Chaetoceros* spp. has been reported to show antioxidative and anti-inflammatory activities (Soontornchaiboon et al., 2012; Ahmed et al., 2014; Kim H. et al., 2019; Kim et al., 2020).

#### Neoxanthin

Neoxanthin (NX), a pigment in spinach, is also available in microalgae. The antioxidative property of NX has been reported in *Scenedesmus* sp*.*, *Chlorella* sp. and *Tetraselmis suecica* (Patias et al., 2017; Sansone et al., 2017). However, NX can also be isolated from *Chlorella vulgaris*, *C. protothecoides*, *Ankistrodesmus gracilis*, *Scenedesmus quadricauda*, *Neochloris oleoabundans*, *Chlorella pyrenoidosa*, *Botryococcus braunii*, *Nephroselmis pyriformis* (Tonegawa et al., 1998; Inbaraj et al., 2006; Magnusson et al., 2008; Chue et al., 2012).

NX has been reported to show anticancer activity against human prostate carcinoma and also responsible for the apoptosis in these cancer cells (Terasaki et al., 2007; Kotake-Nara et al., 2005; Kotake-Nara et al., 2001). In an animal model, NX exhibited anti-initiation activity and also hindered the promotion stage in tumor cells which was revealed through a two-step carcinogenesis study (Lin et al., 1995).

#### Siphonaxanthin

Ketocarotenoid siphonaxanthin (SPX) has been predominately found in microalgae and reported to show better anti-proliferative and anti-angiogenic activity than FX (Sugawara et al., 2014). For instance, SPX from green microalgae *Codium fragile* exhibited apoptosis in human leukemia cells through TRAIL induction with the augmentation of GADD45a and DR5 expression and reduced Bcl-2 and thus showed more effective anticancer property compared to FX (Ganesan et al., 2011). Similarly, this SPX displayed *ex vivo* antiangiogenic activity as well (Ganesan et al., 2010).

#### Cryptoxanthin

Cryptoxanthin is available in many microalgae like *C. vulgaris*, *S. obliquus*, *Aphanothece microscopica Nageli*, *C. pyrenoidosa*, *C. zofingiensi*, *Chlamydomonas planctogloea*, *Selenastrum bibraianum*, *Coelastrum sphaericum*, *Parachlorella kessleri*, *Mougeotia* sp*.*, *S. platensis*, and *P. cruentum* (Jaime et al., 2005; Inbaraj et al., 2006; Patias et al., 2017; Di Lena et al., 2019; Soares et al., 2019). β-Cryptoxanthin obtained from *Cyanophora paradoxa* exerted cytotoxicity against human skin, breast, and lung cancer cells (Baudelet et al., 2013).

β-Cryptoxanthin blocks gastric cancer cells at the G0/G1 phase and induces apoptosis through caspase activation and Cyt C release (Gao et al., 2019). It also displayed anticancer property and apoptosis in HeLa cells (Gansukh et al., 2019). When combined with oxaliplatin, β-cryptoxanthin increased the potency of this chemotherapeutic drug and reduced its toxicity in colon carcinoma (Millán et al., 2015). Moreover, β-Cryptoxanthin hindered lung carcinoma both *in vitro* and *in vivo* experiments (Lian et al., 2006; Iskandar et al., 2016).

### Fatty Acids

Omega-3 polyunsaturated fatty acids, mainly consisting of EPA, DHA as well as α-linolenic acid, is found predominately in fish oil, various plant sources (flaxseed, kiwifruit, chia), and in microalgae, which is effective in the treatment of a different form of cancers such as, breast, colorectal, prostate, ovarian, renal, liver, lung and some other types of cancer (Ashfaq et al., 2019). Microalgal fatty acids are frequently used as fish feed and also as a dietary supplement. EPA has been found in larger amounts in *Chlorella minutissima,* while α-linolenic acid in *H. pluvialis* and *T. suecica* (Rosa et al., 2005). In a study, DHA has been reported to be found in a high amount from Australian microalgae species *Heterocapsa niei* (Mansour et al., 2005). However, EPA and DHA are also obtained from *Phaeodactylum* sp., *Thalassiosira* sp*.*, *Skeletonema* sp*.*, *Cryptomonas* sp*.*, *Tetraselmis* sp., *Isochrysis* sp*.*, *Nannochloropsis* sp., *Porphyridium* sp., *Chaetoceros* sp. (Ryckebosch et al., 2012).

It has been reported that Omega-3 fatty acid supplementation with standard neoadjuvant cyclophosphamide, doxorubicin, and fluorouracil (CAF) chemotherapy and mastectomy improves overall survival and progression-free survival of locally advanced breast cancer patients, through decreasing expression levels of Ki-67 and VEGF leading to inhibition of proliferation and angiogenesis (Darwito et al., 2019). Higher intake of marine ω-3 polyunsaturated fatty acids (MO3PUFA) intake improves survival among stage III colon cancer patients with wild-type KRAS proto-oncogene and deficient DNA mismatch repair, which are responsible for tumor proliferation and survival (Song et al., 2019). Besides, co-supplementation of vitamin D and omega-3 fatty acids significantly reduces inflammatory biomarkers (TNF-a, IL-1b, IL-6, IL-8) and tumor marker, carcinoembryonic antigen in colorectal cancer patients (Haidari et al., 2020). It has been reported that omega-3 supplements can reduce cancer-related fatigue (CRF) in cancer patients under chemotherapy (Ansari et al., 2019). Though omega-3 polyunsaturated fatty acids (O3-PUFA) are widely known for reducing cancer-related fatigue, O6-PUFAs have been documented to significantly reduce CRF compared with O3-PUFA among breast cancer survivors (Peppone et al., 2019).

### Sterols

Microalgal is considered as an alternative source of producing some valuable commercial sterols like, β-sitosterol, stigmasterol, ergosterol, campesterol, and brassicasterol which have pharmaceuticals importance (Randhir et al., 2020). Sterols are found in *Chlorella* sp., *Chlamydomonas* sp., *Scenedesmus* sp., *Ankistrodesmus* sp., *Nannochloropsis limnetica*, *Stephanodiscus hantzschii*, *Gomphonema parvulum*, *Cyclotella meneghiniana*, *Cryptomonas* sp., *Monoraphidium* sp. (Martin-Creuzburg and Merkel, 2016). Along with antioxidative activity, microalgal sterols can show antitumor activity. A sterol-containing fraction of *Nannochloropsis oculate* exhibited anticancer property against human blood, lung, liver, and colon cancer cells (Sanjeewa et al., 2016). Similarly, fatty acid fractions of *Nannochloropsis salina* also showed cytotoxicity against breast cancer cells (Sayegh et al., 2016). Moreover, fatty acids from *S. maxima* have also been reported to show anticancer activity against breast cancer (Elkhateeb et al., 2020).

Sterols can stop tumor growth, metastasis, angiogenesis, and induce apoptosis through caspase-3 activation, Bax/Bcl2 enhancement, or blood cholesterol reduction (Ramprasath and Awad, 2015). Dietary intake of phytosterol can minimize the risk of cancer. For instance, β-sitosterol intake can hinder tumor growth in the human colon, lung, liver, prostate, and breast cancer cells (Jiang et al., 2019).

### Polysaccharides

Microalgae is an excellent reservoir of polysaccharides that has different bioactivity, especially anti-inflammatory, antioxidant and anticancer. For instance, *C. stigmatophora* and *P. tricornutum* can produce polysaccharide extract with anti-inflammatory activity (Guzmán et al., 2003). Polysaccharides obtained from *Tetraselmis* spp., *Pavlova viridis*, *Sarcinochrysis marina*, *Porphyridium* sp. exhibited significant antioxidant activity revealed through antioxidant assays (Tannin-Spitz et al., 2005; Sun et al., 2014; Amna Kashif et al., 2018). In addition, polysaccharide extract of *I. galbana* and *N. oculata* has the antioxidant capacity and antiproliferative activity against HeLa cells (Hafsa et al., 2017). Nostoglycan, a polysaccharide isolated from *Nostoc sphaeroides* has been reported to give protection from oxidative stress, and also to stop the growth of lung cancer cells as well as to promote apoptosis through activation of the caspase-3 pathway (Li et al., 2018). Moreover, polysaccharide fraction of *P. viridis* displayed *in vivo* antitumor property (Sun et al., 2016).

An investigation on the exopolysaccharide-producing microalgae and cyanobacteria revealed that forty-five out of 166 strains were exopolysaccharide producers (Gaignard et al., 2019). *Graesiella* sp., isolated from Tunisian hot spring, possess EPS that have antioxidant activity and show cytotoxicity against human liver and colon cancer cells (Trabelsi et al., 2016). Similarly, *C. pyrenoidosa*, *Chlorococcum* sp., and *Scenedesmus* sp. produce EPS exhibiting antioxidative properties that also have the potential to kill human colon cancer cells (Zhang et al., 2019). On the other hand, sulfated polysaccharides (sPS) with antioxidant activity are extracted from *Navicula* sp. (Fimbres-Olivarria et al., 2018). sPS from *Tribonema* sp. showed antiproliferative and apoptosis in human hepatic carcinoma (Chen et al., 2019). *P. cruentum* having sPS showed *in vitro* and *in vivo* antitumor activity (Gardeva et al., 2009).

### Phycobiliproteins and Peptides

Phycobiliproteins, mainly composed of, phycocyanin, allophycocyanin, phycoerythrin phycoerythrocyanin, are light-harvesting colored protein found predominately in cyanobacteria and also in red algae. Phycobiliproteins have different bioactivities like, antioxidant, anti-inflammatory, anticancer, and others (Pagels et al., 2019). Phycocyanin (PC) plays a protective role against oxidative damage and exerts anticancer activity against different cancers. *Arthrospira platensis* produces PC which shows antioxidant activity revealed through DPPH assay (Pan-utai and Iamtham, 2019). PC isolated from *Porphyra yezoensis* exerted anticancer activity against human melanoma and laryngeal cancer cells in a dose-dependent way (Zhang et al., 2011). PC can block cell cycle at G0/G1 or G2/M phase and induce apoptosis through caspase 3 or 9 activations, reduction of Bcl-2/Bax, COX-2, p-ERK, PEG2, cyclin D1, and CDK4, DNA fragmentation, Cyt c release, ROS generation, reduction of NF-κB, Fas, p53, ICAM-1, CD44, Chromatin condensation. Moreover, PC also downregulates the genes involved in metastasis and angiogenesis. Besides, PC can promote autophagy through blocking Akt/mTOR/p70S6K pathways. Furthermore, PC can enhance the efficacy of chemotherapeutic drugs like doxorubicin, topotecan, betaine, when administered combinedly (Jiang et al., 2017).

Apart from these phycobiliproteins, microalgae also produce protein products, like whole-cell protein, protein hydrolysates, protein concentrates, and peptides which have different biological activities (Soto-Sierra et al., 2018). Microalgal peptides isolated from *S. maxima*, *S. obliquus,* and *T. suecica* have been reported to exert anti-inflammatory, antioxidant, and antimicrobial activity, respectively (Vo et al., 2013; Montone et al., 2018; Guzmán et al., 2019).

### Amino Acids

There is evidence that cancer is related to the interference in amino acid kinetics, which is indicated by an imbalance between plasma amino acids and a higher rate of whole-body turnover of protein and muscle protein breakdown, thus leads to muscle damage. Therefore, increased amino acid supplementation is recommended to promote the synthesis of muscle protein (van der Meij et al., 2019). Supplementation with branched-chain amino acids (BCAA) can control protein synthesis by triggering the mTORC1 pathway which promotes muscle protein balance. Amino acids like arginine and glutamine improve nutritional status in cancer patients undergoing surgery, chemotherapy, and radiotherapy by minimizing inflammation (Soares et al., 2020). In NSCLC, AAs suppress inflammation by increasing the number of CD4^+^ T cells and thus, improve immune status among patients receiving chemotherapy (Liu et al., 2018). However, Brown (1991) stated the presence of all 20 amino acids in 16 microalgae species, where aspartate and glutamate were the most abundant amino acids found in those microalgae. Lim et al., (2018) reported six dinoflagellates having 18 amino acids and glutamic acid was in the highest amount in all species. Additionally, leucine, alanine, valine, and glycine are found to be produced in higher amounts in *C. sorokiniana* and *C. vulgaris* (Ballesteros-Torres et al., 2019).

Mycosporine-like amino acids (MAAs) with the antioxidant property are also commonly found in microalgae. Xiong et al., (1999), reported the presence of five MAAs in *Scenedesmus* sp. and *C. sorokiniana* (Xiong et al., 1999). Llewellyn and Airs (2010) assessed 33 microalgae species and found six MAAs isolated from these microalgae. Among these microalgae, *Glenodinuim foliaceum* was the most prolific producer of MAAs, while shinorine was the most common MAA (Llewellyn and Airs, 2010).

### Minerals

Marine microalgae *P. tricornutum*, *T. chuii,* and *N. granulate* have macro minerals (Ca, P, Mg, K, Na, S) and microminerals (Cu, Fe, Mn, Se, Zn), while *Botryococcus braunii* and *Porphyridium aerugineum* possess all these minerals except Se (Fox and Zimba, 2018). Additionally, *C. ellipsoidea* contains major elements like Na, Mg, Al, K, Ca, Mn, Fe, Cu, and Zn (Kim et al., 2001). Moreover, cookies made from *Spirulina* and *Chlorella* are found high in Se content along with some other minerals Na, Mg, and P (Uribe-Wandurraga et al., 2020).

It has been reported that higher intake of calcium, magnesium, manganese, zinc, selenium, potassium, and iodine intakes, combined with lower intake of iron, copper, phosphorus, and sodium intake can reduce the risk of colorectal cancer incident in postmenopausal women (Swaminath et al., 2019). Supplementation of antioxidants multivitamin and mineral (AMM) protect cancer patients from radiotherapy or chemotherapy-induced oxidative stress, which is indicated by depletion of oxidative stress markers such as MDA and nitric oxide, and restores the endogenous and exogenous antioxidants (SOD, GPx, Vitamin C and Vitamin E) and essential trace element levels (zinc, copper, and selenium), as well (Patil and More, 2020). Moreover, a high daily intake of selenium is protective against cancer, though the effects vary with different cancers (Kuria et al., 2020).

### Coenzyme Q

Coenzyme Q (CoQ10), also known as ubiquinone, is a naturally occurring ubiquitous compound and also an important cofactor in oxidative phosphorylation in mitochondria and associated with cellular energy (ATP) production (Raizner, 2019). Microalgae *Porphyridium purpureum* has been claimed to produce CoQ10, as well as there is also evidence of the presence of CoQ10 in *C. pyrenoidosa* (Klein et al., 2011). Additionally, freeze-dried biomass of *I. galbana* showed a high amount of CoQ10 (Matos et al., 2019).

CoQ10 in combination with alpha-lipoic acid (ALA) prevent cisplatin-induced nephrotoxicity (Khalifa et al., 2020). It has been claimed that coenzyme Q10 inhibits human colon cancer (HCT116) cells through increased ROS and nitric oxide production, while regulating the increased expression of apoptosis-related genes and decreased expression of the anti-apoptotic gene, Bcl2 (Jang et al., 2017). A standard dose of 300 mg/day for 3 months of coenzyme Q10 supplementation has been proposed which can significantly increase antioxidant enzymes activities (SOD, CAT, and GPx) and decreases the levels of inflammatory markers in hepatocellular carcinoma patients after surgery (Liu et al., 2016). On the other hand, it has been observed that high proportion of patients with oral cancer has low ubiquinone and this deficiency is related to high risk of central obesity, hypertriglyceridemia, and metabolic syndrome (Chan et al., 2020). Similar deficiency is often observed in breast cancer also, where supplementation with CoQ10 has been suggested to reduce the adverse effects (Tafazoli, 2017).

## Seaweeds as a Potential Source of Antioxidants

Seaweeds are an important part of Asian cuisine and are rich in pharmaceutically important bioactive compounds. Seaweed antioxidants comprise mainly carotenoids, polyphenols, phycobilin (phycoerythrin and phycocyanin), sulfated polysaccharide, vitamin (A, C) (Cornish and Garbary, 2011). Sulfated polysaccharides and polyphenols from seaweed are not similar to microalgae. Carrageenans, fucoidans, ulvan, and porphyran are the most studied seaweed or macroalgal sulfated polysaccharides that have antioxidant and anticancer activity. Moreover, macroalgae also have non-sulfated polysaccharides like alginic acid, laminarin possessing antioxidative and antitumor properties (Venugopal, 2019). In the case of polyphenolic compounds, the presence of phlorotannins, tetraphloretol, fucophlorethol, eckol, difucol, fucodiphlorethol, phloroglucinol, diphlorethol have been reported from macroalgae (Mekinić et al., 2019). Among all the antioxidant-rich phenolic compounds, phlorotannins, are widely found in macroalgae, especially in brown algae (Montero et al., 2017). Fatty acids from *Laurencia papillosa* (red alga), sulfated polysaccharides from *Pterocladia capillacea,* meroterpenoids like sargachromanol, sargahydroquinoic and sargaquinoic acid from *Sargassum serratifolium,* sesquiterpenoids (isozonarol) from *Dictyopteris undulata* (brown alga) have been reported to exert high antioxidant property (Fleita et al., 2015; Kumagai et al., 2018; Omar et al., 2018; Lim et al., 2019). Besides these, a range of edible seaweeds with antioxidative properties is consumed globally (Table 2).

**TABLE 2:** List of edible macroalgae and their antioxidant capacity.

Macroalgae	Common name	Antioxidant components	Antioxidant activity (*in vitro*)	References
*Laminaria japonica*	Kombu	Polysaccharides	ORAC: 1,247.22 mmol Trolox/g	(Cui et al., 2016)
*Enteromorpha prolifera*	Aonori	Polysaccharides	DPPH: 4.49 mg/ml (IC_50_)	(Xu et al., 2016)
*Porphyra yezoensis*	Nori	Porphyran (sulfated polysaccharide)	OH-radical scavenging: 32.7 μg/ml (IC_50_)	(Isaka et al., 2015)
*Undaria pinnatifida*	Wakame	Phlorotannin	MTT: 40 μg/ml	(Dong et al., 2019)
*Hizikia fusiforme*	Hiziki	Fucoidan	DPPH: 0.55 ± 0.06 mg/ml (IC_50_)	(Wang et al., 2020)
*Chondrus crispus*	Irish moss	Carotenoids	DPPH: 477.73 ± 35.66 µmol Trolox/Kg	(Pina et al., 2014)
*Cladosiphon okamuranus*	Mozuku	Fucoxanthin	DPPH: 1–10 µM	(Mise et al., 2011)
*Caulerpa lentillifera*	Sea grapes	Fatty acids	DPPH: 0.65 ± 0.03 mg/ml (EC_50_)	(Yap et al., 2019)
*Palmaria palmata*	Dulse	EPA (Polyunsaturated fatty acid)	DPPH: 171 ± 19.8 μg/ml (IC_30_)	(Lopes et al., 2019)
*Gracilaria* spp.	Ogonori	Polysaccharide	ABTS: 813.42 ± 194.58 μmol TE/100 g	(Torres et al., 2019)
*Ulva lactuca*	Sea lettuce	Ulvan (sulphated polysaccharide)	DPPH: 13.56 μg/ml (IC_50_)	(Yaich et al., 2017)
*Alaria esculenta*	Winged kelp	Phlorotannin	DPPH: 45.56 ± 5.09 mg/ml (IC_50_)	(Liu et al., 2017)
*Eisenia bicyclis*	Arame	Phenolics	ACW: 7.53 µmol AA/g	(Machu et al., 2015)

## Limitations on Using Antioxidants in Cancer Therapy

Dietary antioxidant supplements can act as a “double-edged sword” in cancer treatment due to their ability to kill cancer cells or to protect them (Favre, 2019). A high daily intake of nutraceutical supplementation may not be safe and may have toxic side effects. Therefore, it is necessary to differentiate the prophylactic dose from the therapeutic dose. A prophylactic dose protects healthy cells and tumor cells, while a therapeutic dose inhibits the growth of only cancer cells. (Calvani et al., 2020). In some cases, low concentrations of free radicals because of the high administration of antioxidant supplementation may promote the proliferation of neoplastic cells rather than interrupting it, thus causing cancer development (Valko et al., 2007). Similarly, herbal supplements are likely to carry a greater risk of pharmacokinetic (PK) interaction with chemotherapy agents compared with vitamin, mineral, and other supplements, which may decrease the efficacy of therapy or create an adverse effect (Luo and Asher, 2018).

The potential harmful or beneficial effect of an antioxidant often depends on its concentration, the presence of other antioxidants, and the concentration of endogenous antioxidants. Many antioxidants interact with the synergic effect with other antioxidants present in the formulation, which is known as “sparing effects.” Administration of a mixture of antioxidants exerts a higher biological effect due to their synergistic activity in various phases, which is more beneficial than a high amount of a single antioxidant (van Breda and de Kok, 2018).

## Conclusion and Future Directions

Over the last few decades, there have been several *in vitro* and *in vivo* studies regarding the antioxidant therapies which have shown that daily intake of a specific dosage of antioxidant nutraceuticals is inversely related to cancer risk as well as enhances the treatment efficacy, nonetheless, randomized clinical trials have shown mixed results which are considered as a real conundrum for the extensive use of antioxidant supplements in cancer therapy. These inconsistent outcomes can be directed by several factors, such as dose, synergism, the bioavailability of antioxidants used, patients’ health status, type of cancer, lifestyle, tendency to supplement intake, and the duration of studies with other variables involved. Therefore, more controlled and well-defined clinical trials with newer approaches need to be conducted to accomplish a safe and effective antioxidant supplement system in cancer treatment. Likewise, there is a need for extensive research to explore novel antioxidant molecules from algae, and their purification strategies as well as *in vivo* investigations should be prioritized. More studies are needed to explore the actual antioxidant compounds present in several organic and aqueous extracts that have already shown *in vitro* antioxidant as well as anticancer activities, and to investigate their mechanism of action on the cellular system and their capability to potentiates chemotherapeutic drugs.
